# Androgen‐type 2 innate lymphoid cells‐dendritic cell axis modulates sex‐associated differences in skin immune responses

**DOI:** 10.1002/mco2.732

**Published:** 2024-09-15

**Authors:** Shi‐Jun He, Jian‐Ping Zuo, Ze‐Min Lin

**Affiliations:** ^1^ Innovation Research Institute of Traditional Chinese Medicine Shanghai University of Traditional Chinese Medicine Shanghai China; ^2^ Laboratory of Immunopharmacology, State Key Laboratory of Drug Research Shanghai Institute of Materia Medica, Chinese Academy of Sciences Shanghai China; ^3^ School of Pharmacy University of Chinese Academy of Sciences Beijing China

1

A recent study by Chi and colleagues in *Science* identified skin type 2 innate lymphoid cells (ILC2s) as crucial for maintaining skin dendritic cell (DC) network homeostasis through cytokine production.^1^ Their findings reveal that the interplay between sex hormones and the microbiota shapes tissue immune set points and DC network strength, with hormones influencing local immunity and the microbiota modulating its intensity.

Differences in the immune systems of females and males contribute to observed sexual dimorphisms in susceptibility to a range of diseases, including cancers, autoimmune diseases, allergies, and infectious diseases, including coronavirus disease 2019, particularly in barrier tissues which are primary sites for infections and are regulated by a complex microbial community. Laffont et al. first identified the role of androgen signaling in regulating ILC2 responses, showing that females have more ILC2s than males.^2^ This disparity arises not from estrogen enhancement in females but from androgen inhibition of ILC2 maintenance and local expansion in males.^2^ Building on this observation, Chi et al. examined the impact of androgen‐mediated regulation of ILC2s on the skin DC network and its subsequent effects on adaptive immune responses.^1^

Acting as nuclear regulators, sex hormone receptors play a pivotal role in fine‐tuning immune responses at the transcriptional level, which in turn influences disease outcomes. In the domain of cancer immunology, these receptors oversee specific pathways in both the innate and adaptive immune systems, providing potential avenues for therapeutic interventions in reproductive cancers. Specifically, androgen receptor (AR) signaling has been linked to the suppression of CD8^+^ T cell function within the tumor microenvironment.^3^ Meanwhile, testosterone, the primary male hormone governing sex differentiation and the development of male sex characteristics, interacts with cytosolic or membrane‐bound ARs to modulate gene transcription either directly or indirectly. ARs are expressed across a variety of cells, including many in developmental stages and some mature immune cells. Further studies across infectious diseases, autoimmunity, and cancer highlight testosterone's direct influence on immune cell development and function, typically leading to immunosuppressive effects.^3^ Additionally, gender disparities in the immune system render males more susceptible to microbial infections and less effective in viral clearance, albeit affording greater protection against autoimmune diseases.^1^ Barrier tissues serve as primary sites for infections and injury and are perpetually inhabited by a diverse microbial community that modulates host defense mechanisms. Despite this, the immune differences specific to each sex in these tissues and their modulation by microbiota are not well characterized. Advancing knowledge on sexual dimorphisms in immune responses may pave the way for the creation of gender‐tailored therapeutic approaches for a multitude of diseases.

In the research by Chi et al., it was demonstrated that sex differences influence the immune responses to microbiota, particularly affecting T helper 17 (Th17) cells and T cell responses to infection.^1^ In both steady‐state conditions and in response to the microbiota, T cell accumulation in the skin was found to be higher in female adult mice than in males. This was evidenced by an increased number of major classical T cell subsets, including Th1 cells, Th17 cells, and interleukin‐17‐producing CD8^+^ T cells, both in the lung and skin of females relative to males. Interestingly, while the study found sex‐specific variations in T cells within the skin of germ‐free mice, such differences were absent in the lung, pointing to a microbiota‐independent impact of sex on skin immunity. Additionally, Chi et al.’s study, which involved conventionalizing adult germ‐free mice with *Staphylococcus epidermidis*, accentuated sex differences, especially in type 17 and Treg cell responses. The results indicate that the microbiota could play a role in shaping the sex‐specific bias towards type 17 and Treg cell immunity in females. In the pre‐pubertal period, when sex hormone levels are low, the distinctions in the lymphoid landscape between females and males are minimal. However, with the onset of sexual maturation and the surge in male sex hormones, these differences become more pronounced. Experiments involving gonad removal demonstrate that male hormones predominantly regulate the observed phenotype. Significantly, castration of male mice restores T cell composition and phenotype to levels akin to females, underscoring the critical role of male hormones in determining lymphoid bias.^1^

Given that DCs are pivotal in tissue immunity and T cell responses to *S. epidermidis*,^4^ Chi et al. explored sexual variations in lymphocyte populations impacting the skin's DC network. They highlight testosterone's role in modulating lymphoid landscapes and maintaining skin DC homeostasis. By crossing Ar^fl/fl^ mice with Il7r^cre^ mice, which impair lymphoid cell response to androgen signaling, they show androgen‐dependent regulation of DC numbers via AR signaling. Single‐cell RNA sequencing reveals significant male‐female differences in ILC2 gene expression linked to immune activation. ILC depletion disrupts the DC network, suggesting their role in DC homeostasis. Adoptive transfer experiments demonstrate ILC2s' ability to control the skin DC network. Injecting ILC2s into Rag2^−/−^γc^−/−^ mice with disrupted DC networks restores cDC1s (CD11c^+^ CD103^+^ CD24^+^ cell) and partially Langerhans cells. ILC2‐derived granulocyte‐macrophage colony‐stimulating factor regulates cDC1 homeostasis, promoting their local accumulation in the skin. Overall, Chi et al. find androgen signaling suppresses ILC2s, reducing DC accumulation and activation in male skin, and attenuating local immunity relative to females. These findings underscore sex hormones, microbiota, and immune cell interplay in shaping tissue immune responses and DC network resilience.

The study by Chi et al. represents a significant advancement in our understanding of sexual dimorphisms in the immune system (Figure 1). Their findings highlight tissue‐specific modulation of immune responses by sex hormones. Specifically, they observed that sex differences predominantly affect skin immunity, consistent with previous studies showing the skin's high number of sex‐biased genes among human tissues. In contrast, no immune differences were evident in the gut, while lung immunity exhibited sex bias only in the presence of microbiota. These results underscore the critical role of local hormone regulation in shaping tissue‐specific immune responses and offer insights into sex differences in disease susceptibility. In addition to the testosterone discussed by Chi et al., researchers have identified a sex bias in the gut microbiome, confirming interactions with other sex hormones such as estrogen, corticosteroids, and progesterone.^5^ However, the molecular mechanisms underlying these host‐microbe interactions at specific barrier sites remain largely unclear. Similarly, the dynamics between effector and regulatory responses needed to maintain or restore host‐microbiota homeostasis are not well understood. Moreover, some human studies have indicated that fluctuations in sex hormones can influence gut microbiota composition. Nevertheless, these findings are often confounded by genetic, environmental, and other factors, resulting in correlations rather than causative relationships between sex hormones and microbiota. To address these gaps, further research is needed to elucidate the precise mechanisms by which sex hormones and microbiota interact to regulate tissue immunity. Identifying the environmental factors influencing these interactions, as well as the timing and dynamics involved, is crucial. Nonetheless, Chi et al.’s research points to potential therapeutic implications for sex‐related diseases, including infectious diseases, autoimmune conditions, and cancers, through strategies like immunotherapy, hormone therapy, and microbiota manipulation. Addressing these complexities will require extensive further investigation.

**FIGURE 1:**
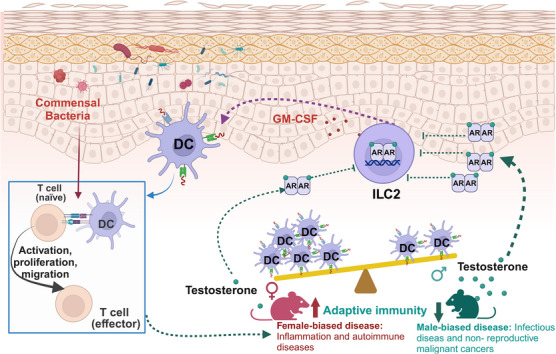
Androgen‐ILC2‐dendritic cell axis modulates sex‐associated differences in skin immune responses. Testosterone, the predominant male hormone, acting via the AR, downregulates cutaneous ILC2s, thereby inducing sex‐specific disparities in both ILC2 and subsequent DC populations. ILC2s may regulate DC homeostasis by producing GM‐CSF, which could subsequently influence T cell activation. Regarding sex‐biased immunity, males exhibit greater susceptibility to infections and non‐reproductive malignancies, whereas females are more prone to inflammation and autoimmune diseases. ILC2s: type 2 innate lymphoid cells; DC: dendritic cell; AR: androgen receptor; GM‐CSF: granulocyte‐macrophage colony‐stimulating factor. The figure was created with BioRender.com.

## AUTHOR CONTRIBUTIONS

**Shi‐Jun He and Jian‐Ping Zuo**: wrote the manuscript. **Ze‐Min Lin and Shi‐Jun He**: drew the figure and approved the final version of the article. All authors have read and approved the final manuscript.

## CONFLICT OF INTEREST STATEMENT

The authors declare no conflict of interest.

## ETHICS STATEMENT

Not applicable.

## Data Availability

Not applicable.
